# Multi-dimensional role of AGEs in periodontitis: from matrix remodeling to neuro-immune crosstalk

**DOI:** 10.3389/fimmu.2025.1643685

**Published:** 2025-11-17

**Authors:** Yu-Lei Dong, Hai Lin, Tao Wen, Zhu-Ling Guo

**Affiliations:** 1 School of Dentistry, Hainan Medical University, Haikou, China; 2 Department of Health Management Center, The First Affiliated Hospital of Hainan Medical University, Haikou, China; 3 Department of Implantology, Stomatological Hospital of Xiamen Medical College, Xiamen, China

**Keywords:** advanced glycation end products (AGEs), periodontitis, extracellular matrix remodeling, neuro-immune regulation, oxidative stress and inflammation

## Abstract

Advanced Glycation End Products (AGEs) are key pathogenic drivers in periodontitis, a chronic inflammatory disease leading to destruction of tooth-supporting tissues. This review synthesizes evidence on the multi-dimensional roles of AGEs, focusing on three core areas: direct modification and degradation of the periodontal extracellular matrix (ECM), induction of a self-perpetuating inflammatory cycle via the Receptor for AGEs (RAGE), and dysregulation of the local neuro-immune axis, an emerging pathogenic frontier. AGEs, which accumulate with age and in metabolic diseases like diabetes, trigger pro-inflammatory signaling (e.g., NF-κB, MAPKs), leading to oxidative stress, cytokine release, and matrix metalloproteinase (MMP) activation. This disrupts ECM homeostasis by suppressing collagen synthesis while promoting its degradation. Notably, specific AGEs like Nϵ-(carboxymethyl)lysine (CML) directly induce osteoblast apoptosis, contributing to alveolar bone loss. A crucial, and increasingly recognized, aspect of AGE pathology is their ability to modulate neuro-immune crosstalk by activating both immune cells and sensory neurons. This creates a complex inflammatory network that exacerbates tissue damage and may contribute to clinical manifestations such as pain and chronic disease. The interplay between systemic AGE load and local production within inflamed periodontal tissues establishes a vicious cycle, making periodontitis a compelling model for studying AGE-driven pathology. Understanding this integrated network reveals novel therapeutic targets aimed at inhibiting AGE formation, blocking RAGE signaling, and modulating downstream inflammatory and neuro-immune pathways to improve periodontal and potentially systemic health.

## 1 Introduction: the converging paths of advanced glycation end products and periodontal destruction

Periodontitis is a chronic inflammatory disease defined by the progressive destruction of tooth-supporting tissues, including the gingiva, periodontal ligament, and alveolar bone (1). Primarily initiated by dysbiotic microbial biofilms, the disease leads to periodontal pocket formation, attachment loss, and ultimately, tooth loss, posing a significant global health burden (2). While microbes are the trigger, the host’s own immune-inflammatory response is the primary engine of tissue destruction (3). In this context, Advanced Glycation End Products (AGEs)—a diverse group of molecules formed non-enzymatically from sugars and proteins—are emerging as critical pathogenic mediators (4). AGEs accumulate with age, are exacerbated by conditions like diabetes mellitus, and can be introduced exogenously through diet and tobacco smoke (5). Their roles in promoting oxidative stress and inflammation are well-established in numerous chronic diseases (6).

The link between diabetes and periodontitis is strong, with periodontitis recognized as a major diabetic complication (7). AGEs serve as a key molecular bridge in this bidirectional relationship (8). Elevated AGE levels are found in the gingival crevicular fluid (GCF) and serum of periodontitis patients, even those without diabetes, pointing to their direct role in periodontal pathology (9, 10). This inherent connection is particularly concerning because both periodontitis and AGE accumulation are considered largely “irreversible” processes (1, 4). This shared feature suggests a compounding effect where persistent molecular damage fuels progressive tissue loss, making established disease exceptionally difficult to manage and underscoring the need for early intervention.

While the roles of AGEs in matrix remodeling and immune activation have been extensively reviewed, their impact on the periodontal neuro-immune axis represents an underexplored, emerging mechanism that deserves focused attention. This review aims to provide a multi-dimensional overview of the pathogenic network orchestrated by AGEs in periodontitis. We will delve into how AGEs: (1) directly alter the periodontal extracellular matrix (ECM); (2) fuel a cycle of inflammation and oxidative stress through their primary receptor, RAGE; and (3) dysregulate local neuro-immune crosstalk, a novel mechanism that may explain key clinical features of periodontitis, such as pain and chronicity. By framing the discussion around this emerging concept, we highlight how periodontitis serves as a model for the complex interplay between systemic factors, local inflammation, and neuro-immune responses, reinforcing the principles of periodontal medicine.

## 2 Advanced glycation end products: formation, diversity, and systemic impact

### 2.1 Definition and formation

Advanced Glycation End Products (AGEs) are a diverse group of molecules formed through a non-enzymatic process known as the Maillard reaction. This reaction begins when a reducing sugar, such as glucose, reacts with the free amino group of a protein, lipid, or nucleic acid to form an unstable Schiff base. This base then rearranges into a more stable, yet still reversible, ketoamine structure known as an Amadori product (e.g., fructoselysine) (4). These early-stage products can then undergo a series of further irreversible reactions—including oxidation, dehydration, and condensation—to form a wide range of irreversible, heterogeneous compounds collectively known as AGEs. This process is significantly accelerated by hyperglycemia and oxidative stress (5). The formation of AGEs can also be driven by highly reactive dicarbonyl compounds like glyoxal, methylglyoxal (MGO), and 3-deoxyglucosone, which are byproducts of glucose metabolism (4). Beyond endogenous formation, the body’s total AGE pool is supplemented by exogenous AGEs from sources like tobacco smoke and thermally processed foods (11).

### 2.2 Chemical diversity and classification

The term “AGEs” encompasses a wide array of structurally varied compounds, with more than 20 distinct AGEs having been identified in human tissues (12). They are broadly classified based on their fluorescent properties. Fluorescent, cross-linking AGEs include well-characterized examples like pentosidine, while non-fluorescent AGEs, which are often more abundant, include Nϵ-(carboxymethyl)lysine (CML) and Nϵ-(carboxyethyl)lysine (CEL) (6). This heterogeneity implies that different AGE species may possess distinct biological activities and pathogenic potentials. Therefore, understanding the specific profile of AGEs in a given condition is crucial for elucidating precise disease mechanisms (13).

### 2.3 Systemic accumulation and pathological consequences

Under normal physiological conditions, a balance exists between the formation of AGEs and their detoxification and excretion, primarily via the kidneys (6). When this balance is disrupted—due to increased formation (e.g., in diabetes), increased intake, or impaired clearance (e.g., in renal insufficiency)—AGEs accumulate in tissues and circulation (14). This accumulation is implicated in a wide range of chronic conditions, contributing to cellular dysfunction and tissue damage by promoting sustained inflammation, inducing oxidative stress, and directly altering protein structure and function by forming cross-links (15). Consequently, higher circulating levels of AGEs are associated with an increased risk of all-cause and cardiovascular mortality (16).

### 2.4 The receptor for AGEs and other AGE receptors

The biological effects of AGEs are largely mediated through their interaction with specific cell surface receptors. The most extensively studied of these is the Receptor for Advanced Glycation End Products (RAGE), a multi-ligand member of the immunoglobulin superfamily expressed on various cells, including endothelial cells, macrophages, lymphocytes, and osteoblasts (17). While RAGE expression is typically low in health, its levels are significantly upregulated in environments rich in its ligands, such as in diabetes and chronic inflammation (5).

**Figure 1:**
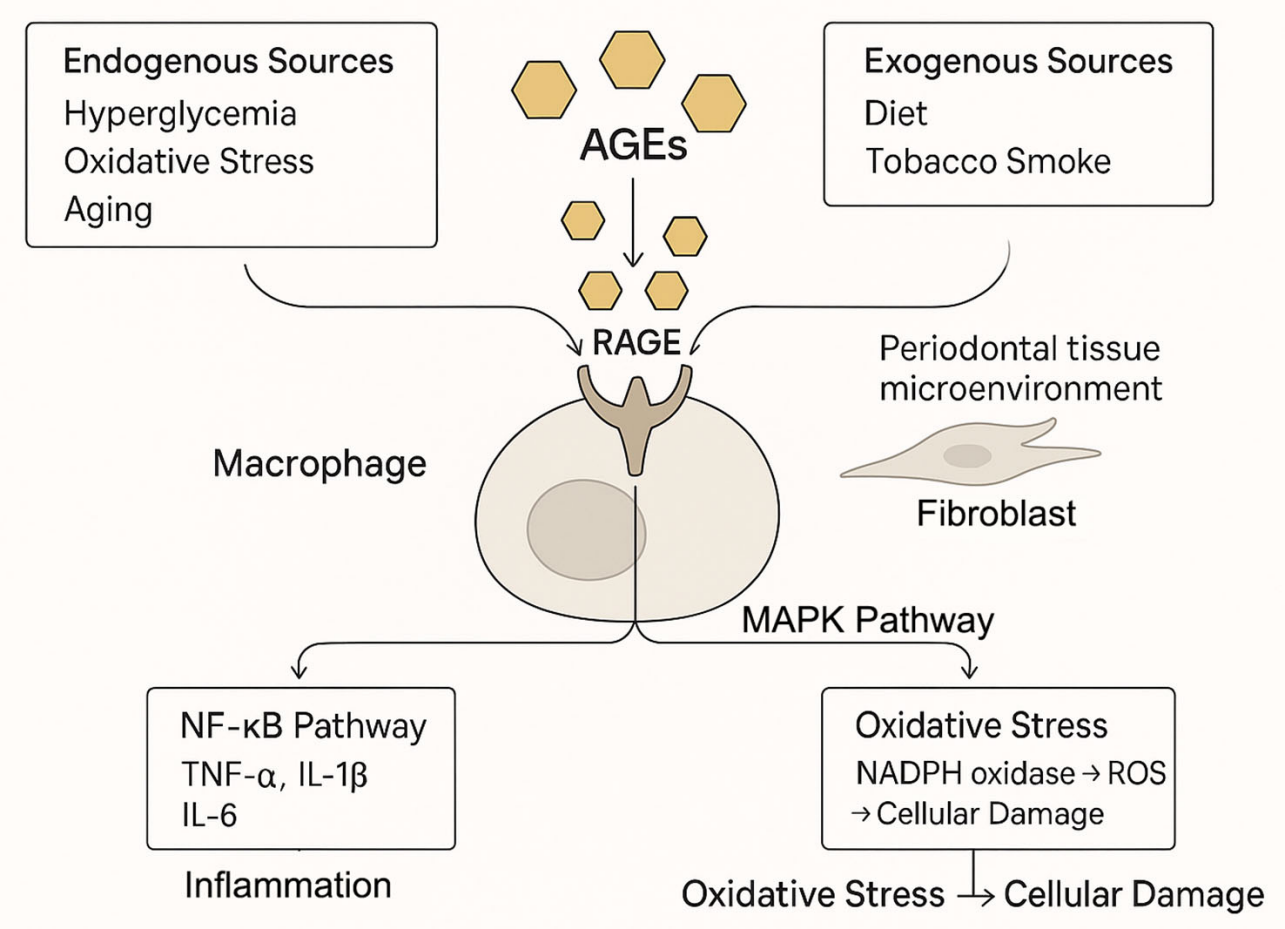
The AGE-RAGE axis as the central engine of periodontal inflammation. Advanced glycation end products (AGEs) accumulate in periodontal tissues from systemic sources (e.g., diabetes, aging) and local production. The binding of AGEs to their receptor (RAGE) on cells like macrophages triggers downstream signaling, including NF-κB and MAPK pathways. This leads to a surge in pro-inflammatory cytokines (e.g., TNF-α, IL-6) and reactive oxygen species (ROS), creating a self-amplifying cycle of inflammation and oxidative stress that drives tissue damage.

The engagement of RAGE by AGEs initiates a cascade of intracellular signaling events that perpetuate cellular dysfunction. Key pathways activated downstream of RAGE include nuclear factor-kappa B (NF-κB) and various mitogen-activated protein kinases (MAPKs) (5). Activation of NF-κB upregulates numerous pro-inflammatory genes, resulting in the production of cytokines (e.g., TNF-α, IL-1β, IL-6) that fuel inflammation (18). Furthermore, AGE-RAGE interaction stimulates NADPH oxidase, leading to increased production of reactive oxygen species (ROS) and heightened oxidative stress (15). This upregulation of RAGE in pathological states creates a detrimental positive feedback loop: AGE accumulation promotes RAGE expression, which in turn sensitizes cells to further AGE-mediated damage, thus amplifying and sustaining inflammatory responses (**Figure 1**).

Beyond the pro-inflammatory RAGE, other receptors are involved in AGE biology. Receptors such as AGE-R1 (OST-48) and galectin-3 are thought to play protective roles by mediating the clearance and detoxification of AGEs (5). Additionally, scavenger receptors like CD36 can bind specific AGEs such as CML, contributing to cellular responses distinct from RAGE signaling (19). The balance between pathogenic signaling via RAGE and the protective functions of these alternative receptors likely plays a critical role in determining susceptibility to AGE-related pathologies.

**Table 1:** Characteristics and pathogenic roles of specific AGEs in periodontitis.

AGE type	Primary precursors	Key receptors	Major pathogenic effects in periodontitis
Nϵ-(carboxymethyl)lysine (CML)	Glyoxal; Oxidized Amadori products	RAGE, CD36	- Induces osteoblast apoptosis via RAGE-MAPK pathway, impairing bone formation (20). - Activates macrophages, promoting pro-inflammatory cytokine release (19). - Alters bone matrix quality, leading to collagen embrittlement (21).
Nϵ-(carboxyethyl)lysine (CEL)	Methylglyoxal (MGO)	Likely RAGE	- Serves as a marker of MGO-driven dicarbonyl stress (22). - Specific mechanisms in periodontitis are largely uncharacterized but presumed to be pro-inflammatory.
Pentosidine	Ribose, other sugars	RAGE	- Forms fluorescent protein cross-links in collagen, increasing stiffness and brittleness (23, 24). - Contributes to age-related decline in tissue mechanical function.

## 3 Periodontitis: a complex inflammatory disease of the tooth-supporting tissues

### 3.1 Definition and etiology

Periodontitis is an inflammatory disease affecting the supporting structures of the teeth, primarily initiated by dysbiotic dental plaque biofilms (2). Key pathogens frequently implicated include Porphyromonas gingivalis, Tannerella forsythia, and Aggregatibacter actinomycetemcomitans (25). However, the critical determinant of tissue destruction is not the microbial burden alone, but the host’s dysregulated immune-inflammatory response to it (26). This response, modulated by genetic and environmental factors (e.g., smoking, diet), can become overly aggressive, leading to collateral damage to the host’s own tissues (27). As potent pro-inflammatory molecules, AGEs are perfectly positioned to fuel this dysregulated host response, tipping the balance towards a destructive phenotype (28).

### 3.2 Pathological features

Clinically, periodontitis is characterized by the progressive destruction of the periodontal ligament and alveolar bone, leading to the formation of periodontal pockets and/or gingival recession (2). If untreated, this can result in tooth mobility and loss (28). Beyond its local effects, periodontitis contributes to systemic inflammation, evidenced by elevated systemic markers like C-reactive protein (CRP) (29). The disease’s prevalence and severity increase with age, a phenomenon linked to “inflammaging”—a chronic, low-grade systemic inflammation that renders tissues more susceptible to damage (30). This complexity explains why management often requires a multi-faceted approach targeting both the microbial insult and the host response (31).

**Table 2:** Impact of AGEs on periodontal cells and extracellular matrix.

Affected component	Key pathological outcomes driven by AGEs
Gingival & PDL Fibroblasts	- Suppressed viability and induction of apoptosis (32). - Decreased Type I & III collagen synthesis (33). - Impaired attachment and migration on glycated matrix, hindering repair (34). - Increased expression of MMP-1, promoting matrix degradation (35).
Osteoblasts	- Induction of apoptosis via RAGE-p38/JNK MAPK pathways, directly impairing bone formation (20). - Attenuated function (e.g., reduced ALP activity) and upregulation of bone formation inhibitors like sclerostin (36).
Collagen Matrix	- Direct damage via non-enzymatic cross-linking (e.g., by pentosidine), leading to increased stiffness and brittleness (24). - Altered charge environment (by CML), leading to defective mineralization (21). - Net degradation due to the combination of suppressed synthesis and increased MMP activity.
Immune Cells (Macrophages, DCs)	- Skewing of macrophages to a pro-inflammatory phenotype via RAGE and CD36 (5, 19). - Impaired migratory capacity and function of dendritic cells (DCs), leading to poor adaptive immune responses (4).

## 4 The AGE-periodontitis nexus: evidence for a pathogenic partnership

### 4.1 Elevated AGEs in periodontal disease: local and systemic evidence

A strong association between AGEs and periodontitis is confirmed by their accumulation in affected individuals. Locally, within the gingival crevicular fluid (GCF)—the inflammatory exudate of the periodontal pocket—AGE levels are significantly elevated in periodontitis patients, with the highest concentrations found in those who also have diabetes (8). More importantly, the impact of periodontitis extends to the systemic AGE load. A key clinical study demonstrated that even in normoglycemic (non-diabetic) individuals, the presence of chronic periodontitis is associated with significantly higher serum AGE levels compared to periodontally healthy controls (10). This crucial finding suggests that the chronic inflammation of periodontitis itself may contribute to the systemic AGE burden, independent of diabetes. This is further supported by immunohistochemical analyses of gingival tissues, which show increased deposition of AGEs and upregulation of their receptor, RAGE, in periodontitis patients regardless of their diabetic status (8). Several studies also report a positive correlation between the severity of periodontal destruction and systemic AGE levels, reinforcing the pathogenic link (37).

**Table 3:** Overview of therapeutic strategies targeting the AGE network in periodontitis.

Therapeutic approach	Example agent(s)	Mechanism & level of evidence
RAGE Antagonism	Soluble RAGE (sRAGE)	Preclinical (Animal Models): Acts as a decoy receptor, blocking AGE-RAGE binding. Shown to reduce bone loss, inflammation, and MMP levels in diabetic mice (7).
Downstream Signal Inhibition	p38 MAPK inhibitors, Notopterol	Preclinical (Animal Models): Inhibit key inflammatory signaling pathways. p38 inhibitors reduced bone loss and cytokine expression in rat models (38). Natural compounds like notopterol show promise (39).
Matrix Degradation Inhibition	Doxycycline	Clinical (Human Use): FDA-approved MMP inhibitor used as an adjunct in periodontitis treatment to reduce collagen breakdown (40).
Antioxidant Therapy	Lycopene, Vitamin C	Clinical (Human Studies): Aim to neutralize ROS. Some studies show adjunctive clinical benefits (e.g., improved BOP, plaque index) (41).
Cytokine Neutralization	Anti-TNF-α (Etanercept)	Preclinical (Animal Models): Biologic agents that neutralize key cytokines. Shown to reduce inflammation and bone loss in periodontitis models (42).
Resolution Promotion	Resolvins (e.g., RvE1)	Preclinical (Animal Models): Actively promote the resolution of inflammation and stimulate tissue regeneration, restoring homeostasis (43, 44).

### 4.2 Periodontitis as an endogenous source of AGEs: a vicious cycle

The AGE-periodontitis relationship is bidirectional; not only do systemic AGEs worsen periodontitis, but the disease process itself generates AGEs, creating a vicious cycle. This local production is driven by both microbial and host factors.

A key breakthrough in this area was the discovery that the periodontal pathogen Tannerella forsythia can directly produce methylglyoxal (MGO), a potent AGE precursor. Mechanistic studies using a genetically engineered MGO-deficient mutant of T. forsythia proved that this metabolic capability is a key virulence factor; the mutant strain failed to cause significant AGE accumulation or bone loss in a mouse model, unlike its wild-type counterpart (45). The clinical relevance of this finding was confirmed in humans, where MGO levels in GCF were found to be nearly 20 times higher at diseased periodontal sites compared to healthy sites (46). This locally produced MGO can then modify host proteins like collagen. Subsequent research has shown that this MGO-modified collagen specifically activates human monocytes through RAGE, triggering the secretion of pro-inflammatory and pro-osteoclastogenic cytokines, thus directly linking a bacterial product to host-mediated tissue destruction (47).

Furthermore, the host inflammatory response itself fuels AGE formation. Activated immune cells in the periodontal lesion release vast amounts of reactive oxygen species (ROS) during the oxidative burst (48). This state of intense oxidative stress creates a highly conducive environment for the chemical reactions that convert Amadori products and other precursors into mature AGEs (8). This evidence paints a clear picture of a self-perpetuating pathogenic loop: systemic AGEs and microbial triggers initiate inflammation, which in turn generates more AGEs locally, amplifying tissue destruction and potentially contributing to the systemic AGE pool (49).

## 5 Molecular specificity of AGEs in periodontal pathogenesis

While often discussed collectively, the chemical diversity of AGEs means that specific molecules may exert distinct biological effects. Understanding this molecular specificity is crucial for developing targeted therapies. Key AGEs implicated in periodontitis include Nϵ-(carboxymethyl)lysine (CML), Nϵ-(carboxyethyl)lysine (CEL), and pentosidine (**Table 1**).

### 5.1 Nϵ-(carboxymethyl)lysine: a key mediator of bone loss

CML is one of the most abundant non-fluorescent AGEs in the human body, formed from the oxidative degradation of Amadori products or the reaction of proteins with glyoxal (5). Its prevalence is particularly high in bone tissue, where it accumulates at levels 40 to 100 times greater than other AGEs like pentosidine, making it highly relevant to the alveolar bone destruction seen in periodontitis (50).

**Figure 2:**
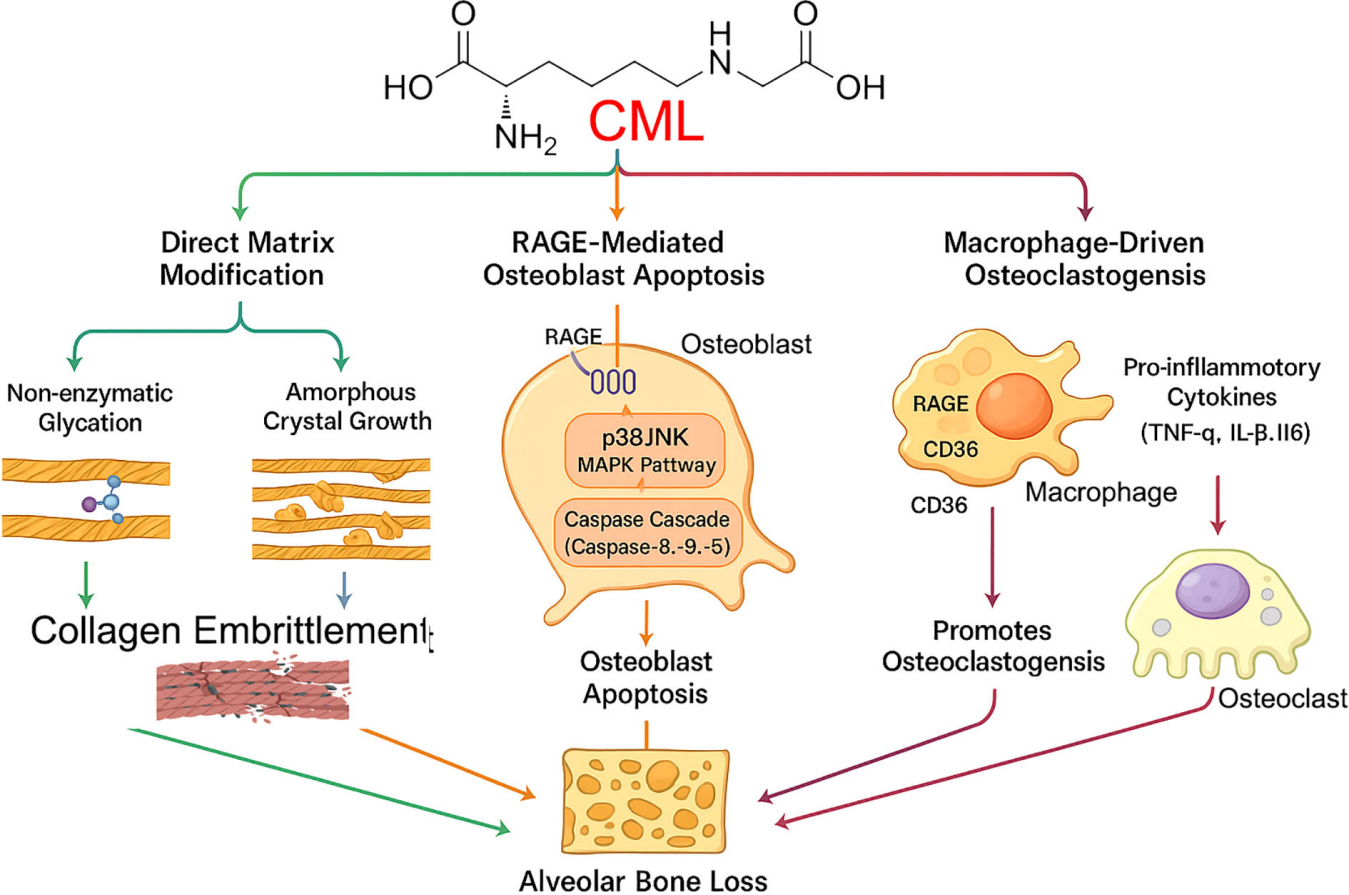
Pathogenic mechanisms of CML in alveolar bone loss. Nϵ-(carboxymethyl)lysine (CML), an advanced glycation end product highly prevalent in bone, orchestrates alveolar bone loss through three convergent pathways. (1) Direct Matrix Modification: CML contributes to non-enzymatic glycation and promotes amorphous crystal growth within the collagen matrix, leading to its embrittlement. (2) RAGE-Mediated Osteoblast Apoptosis: CML binds to the RAGE receptor on osteoblasts, initiating downstream signaling through the p38/JNK MAPK and Caspase Cascade pathways, ultimately inducing osteoblast apoptosis. (3) Macrophage-Driven Osteoclastogenesis: CML activates macrophages via RAGE and CD36 receptors, triggering the release of pro-inflammatory cytokines (TNF-α, IL-1β, IL-6), which in turn promotes the formation and activation of bone-resorbing osteoclasts. All three pathways contribute to the final outcome of alveolar bone loss.

CML exerts its pathogenic effects by interacting with multiple receptors, including RAGE and the scavenger receptor CD36 (19). The mechanisms of CML are illustrated in **Figure 2**, and its detrimental actions in the periodontium are well-documented:

1. Osteoblast Apoptosis: CML, particularly when modifying collagen (CML-collagen), is a potent inducer of apoptosis in osteoblastic cells. This effect is mediated primarily through RAGE and involves the activation of downstream p38 and JNK MAPK pathways, leading to the activation of caspases and programmed cell death (20). By directly killing bone-forming cells, CML significantly impairs bone formation and homeostasis.
2. Macrophage Activation: CML activates macrophages via both RAGE and CD36, promoting a pro-inflammatory phenotype and enhancing lipid uptake (19). This contributes to the sustained inflammation that drives periodontal destruction.
3. Matrix Alteration: Beyond receptor-mediated effects, CML directly alters the quality of the bone matrix. Its formation on collagen changes the local charge environment, leading to aberrant, amorphous hydroxyapatite crystal growth and increased collagen embrittlement, which compromises the biomechanical integrity of alveolar bone (21).

### 5.2 Nϵ-(carboxyethyl)lysine and pentosidine

CEL is another non-fluorescent AGE formed from the reaction of methylglyoxal (MGO) with lysine residues (5). Its levels often parallel those of CML, suggesting it may play a significant role in AGE-related pathologies, though its specific functions in periodontitis are less characterized and represent a knowledge gap (22). Recent studies show that CEL concentrations are increased in psoriatic patients, suggesting its involvement in inflammatory conditions (51).

Pentosidine is a fluorescent, cross-linking AGE that accumulates in tissues with age and in chronic inflammatory diseases (52). Its primary pathogenic role stems from its ability to form non-enzymatic cross-links between collagen fibers (23). This cross-linking increases the stiffness and brittleness of collagenous tissues like the periodontal ligament and bone, making them more susceptible to mechanical failure and impairing normal tissue function (24).

### 5.3 Implications of molecular specificity

The distinct actions of CML, CEL, and pentosidine highlight the importance of molecular specificity. The high prevalence of CML in bone and its potent apoptotic effect on osteoblasts position it as a prime therapeutic target for preventing alveolar bone loss in periodontitis. In contrast, the cross-linking action of pentosidine points to a different mechanism of tissue damage related to biomechanical failure. This suggests that future diagnostic and therapeutic strategies may need to target specific AGEs rather than total AGE load.

## 6 AGE-driven extracellular matrix remodeling in periodontitis

**Figure 3:**
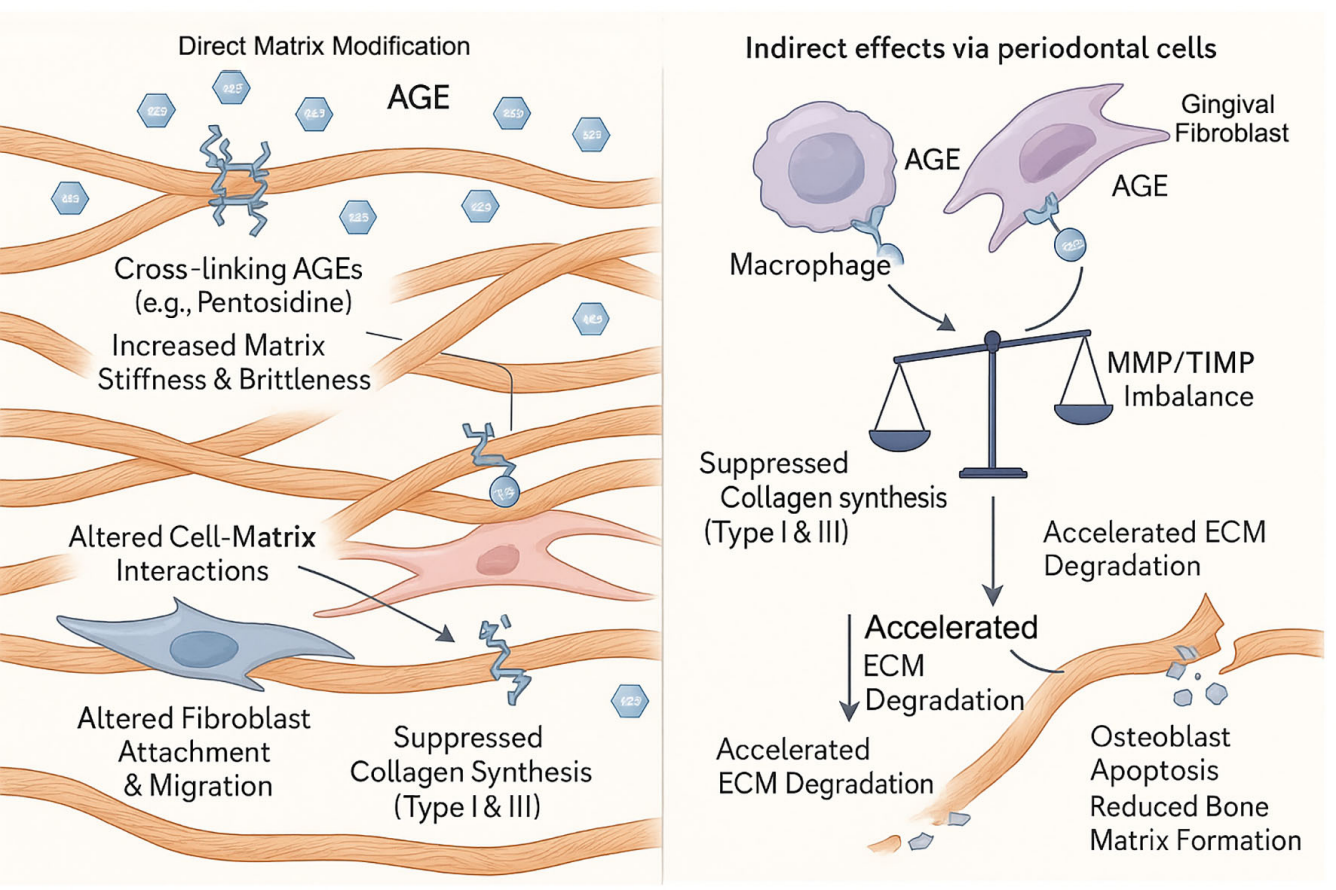
AGE-driven degradation of the periodontal extracellular matrix (ECM). Advanced glycation end products (AGEs) disrupt periodontal ECM homeostasis through a dual-pronged attack. (Left Panel) Direct Matrix Modification: Cross-linking AGEs, such as pentosidine, directly bind to collagen fibrils, increasing matrix stiffness and brittleness. This alteration of the matrix impairs normal cell-matrix interactions, leading to altered fibroblast attachment and migration, and can directly suppress collagen synthesis. (Right Panel) Indirect effects via periodontal cells: AGEs activate resident cells, including macrophages and gingival fibroblasts. This cellular activation creates an MMP/TIMP imbalance and suppresses collagen synthesis (Type I & III), leading to accelerated ECM degradation, osteoblast apoptosis, and reduced bone matrix formation.

The periodontal ECM, rich in collagen, provides structural support and regulates cellular functions. AGEs disrupt ECM homeostasis through a multi-pronged attack that combines direct matrix modification with indirect effects on periodontal cells (**Figure 3**) (**Table 2**).

### 6.1 Direct collagen alterations and impaired cell-matrix interactions

Collagen is a primary target of AGEs. Cross-linking AGEs like pentosidine forms covalent bonds within and between collagen molecules, increasing stiffness and brittleness (24). This compromises the biomechanical properties of the periodontal ligament and alveolar bone. Furthermore, AGE-modified matrix proteins become a dysfunctional scaffold for periodontal cells. Glycated collagen and fibronectin show a reduced capacity to support the attachment and migration of human gingival fibroblasts (HGFs) and periodontal ligament fibroblasts (HPLFs), which is critical for wound healing and tissue maintenance (34). The extracellular matrix undergoes complex changes during aging, with increased cross-linking affecting cellular functions (53).

### 6.2 Dysregulation of matrix synthesis and degradation

AGEs create a net-degradative environment by altering the behavior of resident periodontal cells. This is achieved through two main pathways:

1. Suppression of Synthesis: AGEs suppress the synthesis of new collagen. They have been shown to downregulate the expression of Type I and Type III collagen in HGFs (33) and induce apoptosis in both HPLFs (32) and osteoblasts (20), directly reducing the population of matrix-producing cells. This suppression is mediated through dysregulation of key signaling pathways that control ECM protein expression (54).
2. Enhancement of Degradation: AGEs simultaneously upregulate matrix-degrading enzymes. By activating RAGE, AGEs trigger signaling pathways (NF-κB, MAPKs) that increase the expression of matrix metalloproteinases (MMPs) in periodontal cells (36). For instance, AGEs have been shown to directly induce the expression of MMP-1 (a key collagenase) in HGFs (35). Elevated levels of MMPs, particularly MMP-8 and MMP-9, are consistently found in the saliva and GCF of periodontitis patients (55, 56). The aging process further complicates this matrix degradation, as aged gingival tissues show differential MMP expression profiles compared to younger tissues (37).

This dual-action—suppressing synthesis while promoting degradation—leads to a catastrophic failure of ECM homeostasis, resulting in the progressive tissue destruction characteristic of periodontitis. The importance of maintaining MMP homeostasis is underscored by the fact that MMPs play vital roles in normal tissue turnover, but become destructive when overactivated in periodontitis (57). The imbalance between tissue inhibitors of metalloproteinases (TIMPs) and MMPs contributes significantly to periodontal tissue breakdown (58).

## 7 Neuro-immune dysregulation: an emerging frontier in AGE-mediated pathology

**Figure 4:**
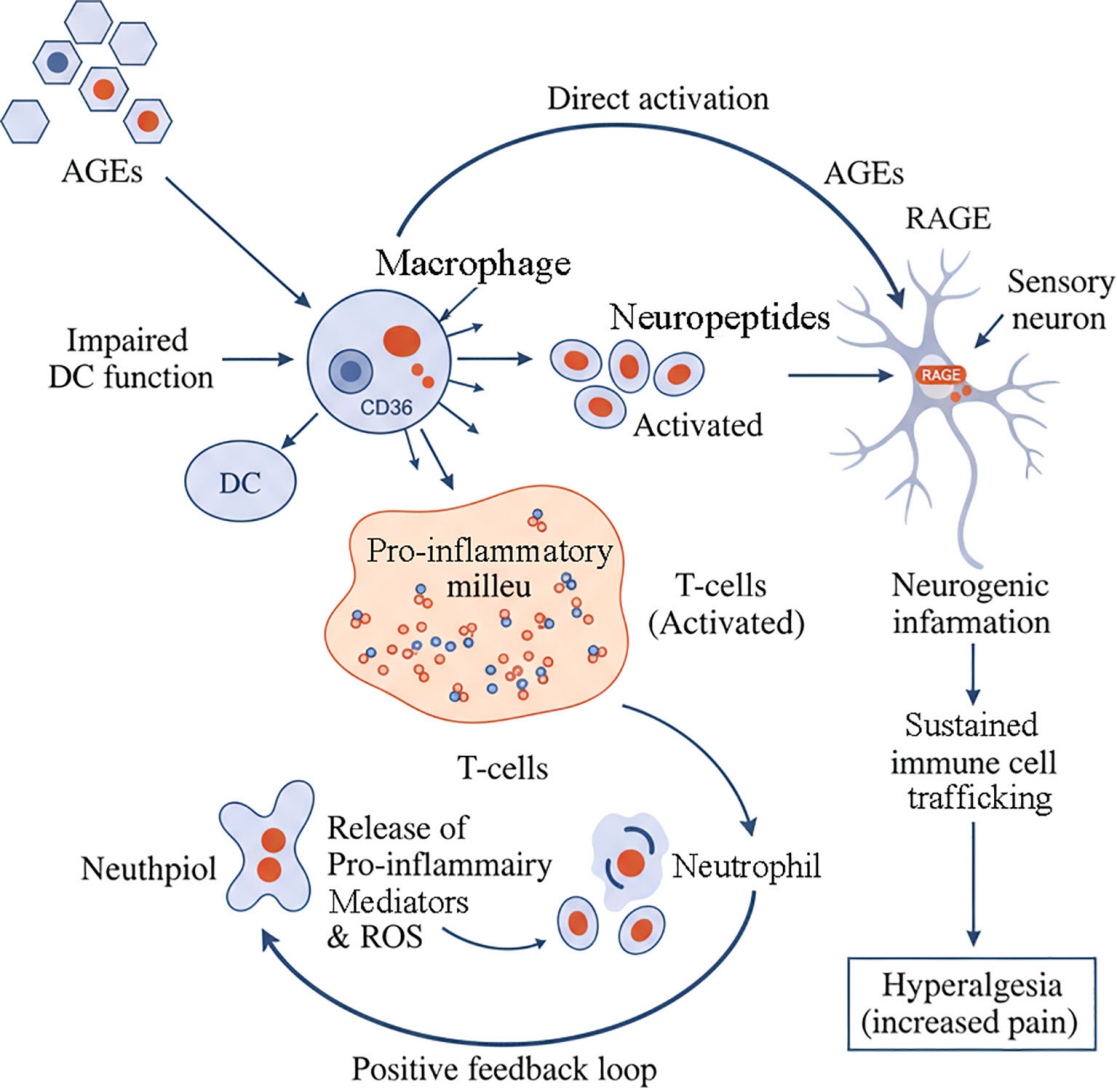
AGE-mediated neuro-immune dysregulation in periodontitis. AGEs orchestrate a complex neuro-immune network that drives chronic inflammation and pain. AGEs directly cause impaired dendritic cell (DC) function and activate macrophages. Macrophages contribute to a pro-inflammatory milieu that activates T-cells. These activated T-cells release neuropeptides, which in turn act on sensory neurons to cause neurogenic inflammation. Concurrently, AGEs can also directly activate RAGE receptors on sensory neurons. This sustained neuronal activation promotes immune cell trafficking and leads to hyperalgesia (increased pain). The pro-inflammatory milieu also activates neutrophils, which release pro-inflammatory mediators and ROS. These mediators feedback to promote the activation of T-cells, establishing a positive feedback loop that perpetuates the inflammatory cycle.

The pathogenic influence of AGEs extends beyond the ECM to involve a complex dysregulation of the local neuro-immune network, representing a novel and clinically relevant frontier in our understanding of periodontitis. The AGE-RAGE axis acts as a central hub, orchestrating pro-inflammatory responses in immune cells and, importantly, modulating the function of sensory neurons within the periodontium (**Figure 4**).

### 7.1 AGE-RAGE signaling: the engine of inflammation and oxidative stress

The binding of AGEs to RAGE on cells like macrophages, fibroblasts, and endothelial cells triggers the activation of NF-κB and MAPK signaling pathways (5). This leads to a surge in the production of pro-inflammatory cytokines (TNF-α, IL-1β, IL-6), chemokines that recruit more immune cells, and ROS that cause oxidative damage (15, 18). This process creates a self-sustaining inflammatory loop that contributes to the chronicity of periodontitis. The aging process further amplifies this inflammatory state, as periodontal tissues become more susceptible to damage with advancing age (59, 60).

### 7.2 Complex immune cell dynamics in periodontitis

The immune response in periodontitis involves multiple cell types that are differentially affected by AGEs:

Macrophages are key players in periodontitis and are highly responsive to AGEs. AGE-RAGE interaction drives them into a pro-inflammatory state, characterized by the secretion of destructive cytokines (5). Specific AGEs like CML can also engage other receptors like CD36 on macrophages, further promoting inflammation (19). The role of macrophages in both periodontal tissue destruction and restoration is complex and age-dependent (48).

Dendritic Cells (DCs) function as professional antigen-presenting cells, and AGEs can impair their function by inhibiting their migration and maturation (4). This dysfunction can lead to an inadequate or inappropriate immune response to periodontal pathogens, increasing disease susceptibility. Aging further compromises DC function, contributing to increased disease susceptibility in older individuals (61).

T Helper Cells, particularly Th17 cells, play crucial roles in periodontitis pathogenesis. These cells are regulated by various factors, including the A20 protein, and their dysregulation contributes to the chronic inflammatory state (62).

B Cells also contribute to periodontal pathogenesis, with their subset distribution being altered in patients with severe periodontitis (63). B cells can promote obesity-associated periodontitis and oral pathogen-associated inflammation (64).

Neutrophils serve both protective and destructive functions in periodontal tissues. While essential for initial pathogen control, their prolonged activation and impaired resolution contribute to tissue damage. The aging process significantly impacts neutrophil functions, contributing to periodontitis progression in elderly patients (65, 66).

### 7.3 Chemokines and age-related changes

The complex network of chemokines plays a crucial role in recruiting and activating immune cells in periodontal tissues. Age-related changes in chemokine expression and function contribute to the increased susceptibility to periodontal disease in older adults (67). Bioinformatics analyses have identified specific periodontitis-related genes and immune cells that are altered in the disease state (68).

### 7.4 Neurogenic inflammation: The AGE-neuron connection

A critical and emerging concept is the role of AGEs in neurogenic inflammation. Periodontal tissues are richly innervated by sensory nerves that not only transmit pain but also actively participate in inflammation by releasing neuropeptides like Substance P (SP) and Calcitonin Gene-Related Peptide (CGRP) (69). The relationship between nociceptor mechanisms and bone remodeling represents an important area of research, particularly in understanding how pain and tissue destruction are interconnected (70).

Crucially, RAGE is expressed on these sensory neurons (71). Emerging evidence indicates that AGE-RAGE interaction can directly activate and sensitize these neurons, potentially contributing to the hyperalgesia (increased pain) often associated with inflammatory conditions (72). This neuronal activation triggers the release of pro-inflammatory mediators from the neurons themselves, which then act on surrounding immune cells, creating a vicious neuro-immune amplification loop (73). This mechanism provides a compelling molecular link between the accumulation of AGEs and key clinical symptoms of periodontitis, such as pain and persistent inflammation, that are often challenging to manage, particularly in patients with high AGE levels, such as those with diabetes (74).

The concept of neuroinflammation as a distal consequence of periodontitis is gaining recognition, highlighting the systemic implications of local periodontal inflammation (75). This neuro-immune crosstalk represents a fundamental aspect of how periodontal inflammation can contribute to systemic diseases (76).

## 8 Integrating the network: a multi-dimensional view of AGEs in periodontitis

The pathogenic role of AGEs in periodontitis is not a linear process but a complex, interconnected network. A central theme is the bidirectional relationship between systemic AGE load and local periodontal pathology. Systemic conditions like diabetes and aging increase the accumulation of AGEs in periodontal tissues (5), where they exacerbate the host response to microbial plaque (8). Conversely, the chronic inflammation of periodontitis, along with the metabolic activity of pathogens like Tannerella forsythia, creates an endogenous source of new AGEs, establishing a vicious cycle (45).

This integrated network demonstrates how AGEs serve as a crucial molecular bridge connecting systemic health factors with localized destructive processes. The core of this network is the AGE-RAGE axis, which, upon activation, triggers a common set of detrimental outcomes across multiple periodontal cell types: increased oxidative stress, heightened pro-inflammatory cytokine production, upregulation of matrix-degrading enzymes (MMPs), and cellular dysfunction or apoptosis (see **Figures 1**-**4**).

The impact of AGEs is significantly amplified in the presence of bacterial components like lipopolysaccharide (LPS) (36), meaning that disease progression is likely to be most aggressive when both high AGE levels and significant bacterial load are present. Furthermore, the emerging neuro-immune component reveals that AGEs can directly activate sensory neurons via RAGE, triggering neurogenic inflammation that crosstalks with the immune system (72). This amplifies local inflammation and may contribute to clinical symptoms like pain and chronicity, offering a more complete picture of how AGEs orchestrate a multi-pronged attack on the periodontium.

## 9 Therapeutic perspectives: targeting the AGE pathogenic network

The multi-dimensional involvement of AGEs in periodontitis offers several potential avenues for therapeutic intervention, from inhibiting their formation to promoting the resolution of inflammation (**Table 3**).

### 9.1 Inhibition of AGE formation and RAGE blockade

Strategies to disrupt the AGE network include inhibiting AGE formation (e.g., with aminoguanidine) or breaking existing cross-links (e.g., with alagebrium), though their specific efficacy in periodontitis requires dedicated research. A more promising approach is targeting the AGE-RAGE axis. Soluble RAGE (sRAGE), which acts as a decoy receptor, has shown significant therapeutic potential. In a preclinical diabetic mouse model, administration of sRAGE dose-dependently reduced alveolar bone loss, decreased pro-inflammatory cytokine and MMP levels in gingival tissues, and lowered gingival AGE accumulation (7, 80). This suggests sRAGE can break the vicious cycle of inflammation and AGE generation. Clinically, serum sRAGE levels negatively correlate with the Bleeding on Probing (BOP) index, a key clinical indicator of periodontal inflammation where a periodontal probe is used to assess bleeding from the gingival sulcus (77). However, translating sRAGE therapy to clinical use faces challenges, including the need to establish optimal dosing, delivery methods (systemic vs. local), and long-term safety. Additional research has shown that RAGE and its ligands play important roles in inflammatory and accelerated periodontal disease associated with diabetes, providing mechanistic insights into therapeutic modalities (78).

### 9.2 Modulation of downstream pathways

Targeting the signaling pathways activated by AGE-RAGE interaction offers another layer of intervention:

**NF-κB and MAPK Inhibitors:** Since NF-κB and MAPKs are key mediators of AGE-induced inflammation, their inhibition is a logical approach. Natural compounds like notopterol have shown promise in inhibiting the NF-κB pathway while activating protective PI3K/AKT/Nrf2 pathways in periodontal tissue (39). In preclinical models, inhibitors of p38 MAPK have been shown to reduce periodontal bone loss and inflammation (38). However, the broad and essential roles of these pathways in normal cellular function raise concerns about the potential for off-target effects with systemic, long-term use.
**MMP Inhibitors:** Sub-antimicrobial doses of doxycycline, an MMP inhibitor, are already FDA-approved for periodontitis treatment, validating this approach (40).
**Antioxidants and Cytokine Inhibitors:** The role of antioxidant enzymes in periodontitis is well-established (79), and antioxidants like lycopene have shown some adjunctive benefit in clinical studies (41). Biologic agents that neutralize specific cytokines like TNF-α or IL-6 have demonstrated efficacy in preclinical periodontitis models but carry the risk and cost associated with systemic immunosuppressive therapies (42).

### 9.3 Promoting resolution of inflammation

An emerging paradigm shift is to move beyond simply inhibiting inflammation and instead actively promoting its resolution. Specialized pro-resolving mediators (SPMs), such as resolvins and lipoxins, orchestrate the termination of inflammation and stimulate tissue regeneration (81). In preclinical studies, topical application of SPMs has been shown to control inflammation, promote bone regeneration, and even reverse microbial dysbiosis (43). This approach is highly promising as it aims to restore homeostasis rather than simply blocking a single pathway, potentially offering a more holistic and safer therapeutic strategy. The resolution of inflammation represents a novel approach to treating periodontal diseases, with significant clinical potential (44).

## 10 Conclusion and future directions

Advanced Glycation End Products are pivotal players in the complex pathogenesis of periodontitis. Their influence is multi-dimensional, spanning direct matrix destruction, profound immune dysregulation, and intricate neuro-immune interactions. The AGE-RAGE axis acts as a central engine, fueling a vicious cycle of chronic inflammation and tissue degradation that is particularly devastating in high-risk populations, such as individuals with diabetes and the elderly.

A key message of this review is the need to look beyond the classical understanding of AGEs and focus on the emerging frontier of neuro-immune crosstalk. The ability of AGEs to directly activate sensory neurons provides a compelling molecular basis for clinical features like pain and chronicity, bridging the gap between molecular pathology and patient experience. This positions periodontitis as an important clinical model for studying the systemic consequences of AGE accumulation and local neuro-inflammatory processes.

The elucidation of this complex network has paved the way for novel host-modulatory therapies. While strategies targeting RAGE, downstream signaling, and oxidative stress have shown promise in preclinical models, significant hurdles remain for clinical translation, including specificity and long-term safety. In this context, therapies based on specialized pro-resolving mediators, which aim to restore homeostasis, represent a particularly exciting path forward.

Future research should focus on: (1) elucidating the specific roles of different AGE species in neuro-immune interactions; (2) conducting robust clinical trials to translate promising preclinical findings into effective human therapies; and (3) developing reliable biomarkers to identify patients who would most benefit from AGE-targeted interventions. A holistic approach that considers the systemic and local impact of AGEs is imperative for future therapeutic innovation in periodontal medicine.
